# Differentiation Therapy of Acute Myeloid Leukemia

**DOI:** 10.3390/cancers3022402

**Published:** 2011-05-16

**Authors:** Elzbieta Gocek, Ewa Marcinkowska

**Affiliations:** Department of Biotechnology, University of Wroclaw, ul Tamka 2, Wroclaw 50-137, Poland; E-Mail: ela@protein.pl (E.G.)

**Keywords:** acute myeloid leukemia, targeted therapy, differentiation, all-trans retinoic acid, acute promyelocytic leukemia, 1,25-dihydroxyvitamin D_3_, vitamin D analogs

## Abstract

Acute Myeloid Leukemia (AML) is a predominant acute leukemia among adults, characterized by accumulation of malignantly transformed immature myeloid precursors. A very attractive way to treat myeloid leukemia, which is now called ‘differentiation therapy’, was proposed as *in vitro* studies have shown that a variety of agents stimulate differentiation of the cell lines isolated from leukemic patients. One of the differentiation-inducing agents, all-trans retinoic acid (ATRA), which can induce granulocytic differentiation in myeloid leukemic cell lines, has been introduced into clinics to treat patients with acute promyelocytic leukemia (APL) in which a PML-RARA fusion protein is generated by a t(15;17)(q22;q12) chromosomal translocation. Because differentiation therapy using ATRA has significantly improved prognosis for patients with APL, many efforts have been made to find alternative differentiating agents. Since 1,25-dihydroxyvitamin D_3_ (1,25D) is capable of inducing *in vitro* monocyte/macrophage differentiation of myeloid leukemic cells, clinical trials have been performed to estimate its potential to treat patients with AML or myelodysplastic syndrome (MDS). Unfortunately therapeutic concentrations of 1,25D can induce potentially fatal systemic hypercalcemia, thus limiting clinical utility of that compound. Attempts to overcome this problem have focused on the synthesis of 1,25D analogs (VDAs) which retain differentiation inducing potential, but lack its hypercalcemic effects. This review aims to discuss current problems and potential solutions in differentiation therapy of AML.

## 1. Introduction

Leukemia is a disease of the blood or bone marrow, which is characterized by increased numbers of abnormal white blood cells. The abnormality of leukemic cells lies in their inhibited differentiation and increased proliferation rate. Leukemia is divided into acute and chronic, and further subdivided into lymphocytic and myeloid [1]. Within these groups further divisions are often necessary. The most heterogeneous group being a group of acute myeloid leukemias (AML) [2]. In leukemias differentiation block occurs in early hematopoietic progenitors, and resulting malignant cells are named blast cells. Acute leukemias are diagnosed either on the basis of presence of over 20% of blasts in the blood or in bone marrow or on the basis of presence of specific cytogenetic or molecular abnormalities [1]. There are more than 200 known chromosome translocations and mutations in leukemic cells of patients diagnosed with AML [3]. Which of these 200 are initial mutations responsible for clonal expansion of abnormal cells, and which are accumulated during the progress of disease, is largely unknown. AML is rare in children, it constitutes 80–85% of acute leukemia in adults, and its incidence increases with age [4]. The progress in understanding molecular background of the disease has led to significant changes in the classification of AML subtypes. Before 2001, the French-American-British (FAB) classification was used, in which AML was divided into eight subtypes (M0—M7), based on the type of cell from which the leukemia developed and on its degree of maturity [5]. In 2001 WHO introduced a more accurate division that incorporates cytogenetic abnormalities and prognostic significance. WHO classification was further revised in 2008 and has classified AML into four main groups: AML with recurrent genetic abnormalities, AML with myelodysplasia-related changes, therapy-related myeloid neoplasms, and AML not otherwise specified [6]. It should be also remembered that the disease is not common. In our country, Poland, with a population of about 38 million people, there are about 1000 cases of AML diagnosed per year [7]. Taking together the numbers and the variety of genetic alterations, it becomes clear that finding of a specific treatment for AML is not an easy task.

## 2. A Paradigm of Targeted Cancer Therapies

The idea of targeted therapies is not new. In his speech at the ceremonial opening of the Georg-Speyer-Haus in September 1906, Paul Ehrlich proposed that drugs should work as “magic bullets” that kill pathogens, and leave normal tissue unaffected [8]. Ehrlich's “magic bullets” were directed towards microorganisms, but later on the idea was adopted for anticancer treatment. Of course, every drug has its target, and most of the currently used anticancer chemotherapeutic drugs target, in a more or less direct manner, synthesis of DNA and cell division. Since cancer cells are not the only ones that need to proliferate in human body, chemotherapeutic drugs are often toxic and cannot be considered as “magic bullets” or in other words, targeted drugs. Therefore targeted anticancer therapies should be based on compounds that interfere with cellular components that are altered or present only in cancer cells [9].

Paradigmatic targeted therapy is embodied by treatment of patients with chronic myeloid leukemia (CML) using Imatinib [10]. Over 90% of CML patients carry chromosomal abnormality called the Philadelphia (Ph) chromosome [11,12]. Therefore the drug that interferes selectively with the tyrosine kinase activity of the fusion protein Bcr-Abl, should be toxic only to CML malignant cells [13]. The introduction of Imatinib (and similar specific inhibitors of Bcr-Abl) has revolutionized treatment of CML patients and increased rates of complete hematological response to 97% and complete cytogenetic response to 85% [14].

The most important difficulty in finding appropriate regimens of targeted therapy for AML patients originates from the above mentioned heterogeneity of the disease. Among 200 known chromosome translocations and mutations in AML, some are more common than others. One of the most common mutations seen in AML is internal tandem duplication in fms-like tyrosine kinase 3 (FLT3-ITD). This mutation is apparent in about 25% of all AML patients and confers unfavorable prognosis [2,15]. There is also another mutation in FLT3 receptor, point mutation in the tyrosine kinase domain (FLT3-TDK), seen in approximately 7–8% of AML patients, with less defined prognostic significance [2,16]. FLT3 receptor tyrosine kinase is normally expressed in immature precursors of myeloid and B-lymphoid lineages [17,18]. FLT3 ligand (FLT3-L) together with other colony stimulating factors and interleukins can stimulate proliferation of hematopoietic cells [19,20]. The two above mentioned mutations result in a ligand-independent activation of the receptor and give survival advantage to blast cells over their normal counterparts [21]. Aberrant FLT3 receptor seemed to be an attractive therapeutic target in AML, so several small molecules FLT3 tyrosine kinase inhibitors (TKI) have been developed and examined *in vitro* and *in vivo*. Eight of them, after successful preclinical screening, have been introduced into clinical trials [22]. The data available at the moment show that TKIs, used as single agents did not fulfill expectations and that the quality of clinical response was unsatisfactory [22].

## 3. Differentiation Therapies for Leukemia

Leukemic cells are inhibited in their hematopoietic differentiation by either genetic abnormalities or by gene expression abnormalities [2]. These cells proliferate rapidly, but often do not express proteins important for function of their normal counterparts. Even though white blood cells counts are high in these patients, the immune functions are lacking. Therefore, finding a method of forced differentiation of leukemic cells always seemed attractive to researchers and clinicians. Differentiation therapy seems to be a particularly attractive solution for AML patients. These patients are often elderly and rapidly progressing disease causes poor tolerability of intensive cytotoxic protocols [1]. Forced differentiation of myloid precursors should, in principle, improve the immune status of patients without massive lysis of blast cells seen in some cytotoxic regimens [23].

It should be remembered that inhibited differentiation may result not only from presence of mutated proteins, but also from epigenetic changes like DNA hypermetylation or aberrant acetylation of histones [9]. On the contrary to the loss of gene function caused by mutations, epigenetic changes can be reversed via pharmacologic inhibition of DNA methyltransferases (DnmT) and histone deacetylases (HDAC). In normal cells, histone acetylation and DNA methylation are maintained in equilibrium, allowing temporal expression of the genes. In leukemic cells, this balance is disturbed, hypermethylation occurs, HDACs are overexpressed, what leads to the transcriptional repression [24]. Therefore, several HDAC and DnmT inhibitors have been under development for the treatment of patients with hematological malignancies, such as AML or MDS, either as monotherapies or in combination with other agents [25-28]. Treatment of leukemia cells with such inhibitors, results in chromatin remodeling that unblocks a set of genes whose transcriptional activation induces cellular differentiation, cell cycle arrest, apoptosis or autophagy [26,29,30]. miRs, small non-coding RNAs 19–25 nucleotides long, provide an additional level of control between proliferation and differentiation [31,32]. They regulate gene expression post-transcriptionally via degradation of target mRNAs or/and via inhibition of protein translation [33,34]. A single miR can control levels of hundreds different target genes. Many miRs have been linked to the specification of hematopoietic cell lineages, and have been found altered by chromosomal translocations associated with leukemia and therefore constitute potential therapeutic targets [35-38].

## 4. New Treatments for Acute Promyelocytic Leukemia as Examples of Differentiation Therapy

Acute Promyelocytic Leukemia (APL) is the first hematological malignancy in which therapeutic approach specifically targeting the underlying molecular lesion has been successfully introduced into clinical practice. APL is a subset of AML characterized by uncontrolled expansion of leukemic blast cells, blocked at promyelocytic stage of hematopoiesis, in the bone marrow [39]. Morphologically, it is classified as a subtype M3 of AML [5], cytogenetically is characterized by a reciprocal translocation between the long arms of chromosomes 15 and 17 [t(15;17)] [40-43]. These aberrations lead to the fusion between promyelocytic leukemia (PML) gene located on chromosome 15q21, and retinoic acid receptor α (RARA) gene from chromosome 17q21, and to the formation of the resultant chimeric oncoprotein PML-RARA [42,44]. The fusion transcript of PML-RARA is detectable in more than 95% of APL patients with t(15;17), and becomes a major player disturbing proper promyelocytic differentiation, as well as a molecular marker for this disease [41,45]. In the remaining minority of patients, alternative fusions of RARA may occur [46-49]. These findings enabled the introduction of all- *trans*-retinoic acid (ATRA) as a differentiation agent for APL treatment.

### 4.1. PML-RARA-Induced Transcriptional Repression in APL Cells

In normal hematopoietic cells, retinoids play multiple physiological roles in maturation and differentiation [50-52]. These compounds function through binding to their receptors (RAR and RXR), which belong to the superfamily of nuclear ligand-activated transcription factors [53,54]. In the absence of a ligand, RARA forms heterodimers with the RXR, binds to the retinoid acid response elements (RARE) in the promoter region of the target genes and recruits co-repressor (CoR) complex. CoR is composed of several proteins, including nuclear receptor co-repressor (NCoR), silencing mediator for retinoid and thyroid hormone receptors (SMRT) [55]. These proteins recruit DNA DnmT1 and DnmT3a and HDAC1 [56-58]. Deacetylated histones cause chromatin condensation and transcriptional repression [56,59]. At physiological concentrations (10^−9^–10^−8^M), ATRA binds to the RARA-RXR hetrodimer and induces dissociation of CoR, interchangeably with association of the co-activators (CoA) complex, containing histone acetyltransferase (HAT). Acetylated histones cause chromatin decondensation and activation of the transcription [56,59].

In APL, RARA heterodimerizes with promyelocytic leukemia (PML) nuclear protein, normally responsible for the formation of the nuclear bodies and regulation of the stem cells self-renewal [60,61]. PML-RARA fusion protein acts as an oncogene, causing enhanced proliferation and inhibited terminal differentiation of the hematopoietic cells. PML-RARA heterodimers act in a dominant negative manner over RARA, and have higher affinity to CoR and HDAC than RARA-RXR, resulting in enhanced hyper-methylation of the DNA [62,63]. Furthermore, PML-RARA oligomerizes in a different manner to RXR-RARA and may not only homodimerize, but also heterodimerize with wild-type PML and RXR [64]. This may cause sequesteration of those proteins and recruiting enzymes in a large complex, which augments transcriptional repression [62,65]. An example of genes repressed by PML-RARA is the one encoding protein p21^Cip1^, what may contribute to the final effect of enhanced proliferation of APL cells [64,66,67]. Heterodimerization with PML-RARA turns off pro-apoptotic and growth inhibiting properties of PML [68,69]. Interfering with the normal function of both, RARA and PML, the fusion protein reveals double dominant negative activity [68]. PML-RARA also binds promyelocytic leukemia zinc finger (PLZF) protein, and affects its functions as a growth suppressor [70]. Moreover, it appears that PML-RARA can enhance pro-proliferative and pro-survival functions of FLT3 [71,72]. It is important to note that PML-RARA often targets PU.1-regulated promoters through both protein-protein interactions, as well as DNA binding via RARE half sites [73]. Genes containing these PML-RARA-targeted promoters are transcriptionally suppressed in APL and most likely constitute a major mechanism of transcriptional repression occurring in APL [73]. Molecular disturbances mentioned above contribute to the blockage of granulocytic differentiation, and constitute the main reason for the inability of ATRA to unblock transcriptional repression at its physiological concentration.

### 4.2. ATRA-Induced Differentiation and Elimination of APL Cells

**Figure 1.**
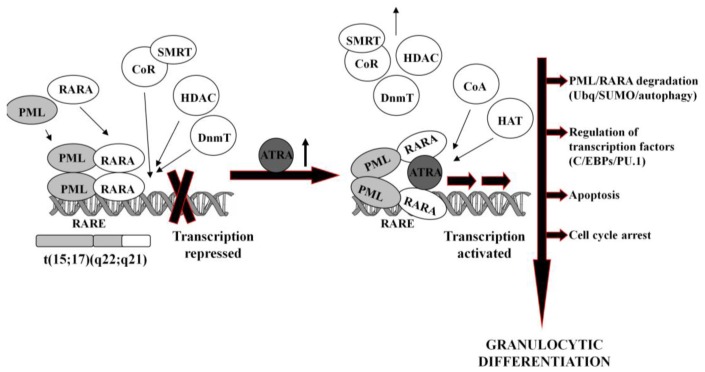
Molecular mechanism of ATRA-induced differentiation of APL cells. In APL cells, PML-RARA hereodimerization leads to the sequestration of CoR complex, DNA hypermethylation and histones deacetylation, resulting in transcriptional repression. In the presence of high-dose of ATRA the CoR is replaced by CoA, allowing transcriptional activation and terminal differentiation of the APL cells into granulocytes. PML-RARA fusion protein undergoes degradation by several ways, including ubiquitination, sumoylation or authophagy. (PML: promyelocytic leukemia; RARA: retinoic acid receptor; RARE: retinoid acid response elements; CoR: co-repressor; SMRT: silencing mediator for retinoid and thyroid hormone receptors; HDAC: histone deacetylase; DnmT: DNA methyltransferases; CoA: co-activators; HAT: histone acetyltransferase; Ubq :ubiquitin; SUMO: sumoylation).

It is generally accepted that at pharmacological concentrations (10^−7^–10^−6^M) ATRA causes conformational changes of PML-RARA, which enable dissociation of the CoR and association of the CoA. As a result, chromatin structure becomes relaxed, transcriptional repression relieved, and APL cells undergo terminal differentiation into granulocytes (Figure 1) [74,75]. It has been reported that several miRs (*i.e.*, miR-223, let-7a) play an important role of a regulatory circuit involving C/EBPα and NFI-A, master regulatory transcription factors that control granulocytic differentiation in ATRA-treated APL cells [36,76]. Moreover, it has been shown that several miRs (*i.e.*, miR-210, miR23a/24-2) which are transcriptionally repressed by the APL-associated PML-RARA oncogene, become activated after treatment with ATRA [77-79].

ATRA-induced degradation of PML-RARA is the basic therapeutic mechanism in APL cells [42,80]. Recent results have revealed several ways leading to the destruction of the fusion oncogene, such as ubiquitination [81], sumoylation [82] or authophagy [83]. PML-RARA degradation is accompanied by the activation or inhibition of a number of various ATRA-response genes. These include transcription factors (for example C/EBPε or PU.1) [73,84], chromatin-regulating factors [63,85], cell cycle regulators [86,87], as well as protein synthesis inhibitors [88].

Effectiveness of ATRA in the treatment of APL is unquestionable. In more than 90% of the patients such a therapy leads to the complete remission [89,90]. Treatment regiments combining ATRA with arsenic trioxide (ASO) further improved curability of the patients, especially these with ATRA-resistance, usually occurring after long-term use of the drug [90,91]. The possible reason for the relapse is that ATRA used as a single agent, in spite of inducing complete remission, is unable to cause complete molecular remission. Therefore a fraction of leukemia initiating cells (LIC) may remain after initial successful ATRA treatment [80]. In contrast, ATRA/ASO combination therapy has been shown to produce final clearance of LICs [80,92]. In leukemic cells ASO supports ATRA-induced differentiation, but this is not enough for eradication of the disease. Even though ASO has been known as a therapeutic agent for ages, its mode of action remained obscure until recently [93]. At present we know that it affects cells in many different ways, one of the most important effects in APL cells is degradation of PML-RARA oncogene, a step necessary for eradication of LICs [94]. As mentioned above, similar effect of PML-RARA degradation may be obtained with ATRA alone, but only when its high intracellular levels are obtained. ATRA/ASO combination therapy synergizes molecular effects of both drugs [92]. At a molecular level ASO targets PML moiety in fusion PML-RARA protein where it triggers formation of arsenic-cysteine bonds that favor protein aggregation. PML-RARA in aggregates undergo ubiquitination, sumoylation and resulting degradation in proteasomes [93]. At a cellular level ASO induces apoptosis, mainly through the mitochondria-mediated intrinsic apoptotic pathway [95].

In its first description APL was considered to be the most malignant form of AML, accompanied by severe bleeding and short survival time, and now it is the most curable one [39,90]. Successful molecular-targeted therapy with ATRA/ASO, has opened a new area in cancer therapy, and raised the possibility that other diseases may be treated by different compounds in a similar way. Since 1,25-dihydroxyvitamin D_3_ (1,25D) is capable of inducing monocytic differentiation of AML cells *in vitro* [96,97], it is one of the best prospects for use in clinical applications [98].

### 4.3. ATRA in Non-APL Subtypes of AML

The possibility to induce differentiation of HL60 cells using ATRA has been known for over 30 years [99]. However, at the time of this discovery HL60 cells were believed to be APL, it only manifested later that they originate from subtype M2 of AML, according to FAB classification [100]. Further studies have shown that there are more non-APL AML cell lines that respond to ATRA. For example in THP-1 cells ATRA up-regulates expression of C/EBPα and β transcription factors [101], which are key regulators in myeloid cell differentiation and also important regulators of ATRA-induced differentiation of APL cells [102]. Such findings stimulated clinical attempts to combine ATRA with chemotherapy for non-APL AML patients. Some early clinical trials have been conducted, but resulting conclusions were confusing. Some trials did not show benefits of combination therapy for patients [103,104], while others did [105]. Therefore a search for more specific differentiation agents for non-APL AML is underway and will be discussed below.

## 5. 1,25D and Its Low-Calcemic Analogs for Differentiation Therapy

The major role of 1,25D in human body is maintenance of calcium/phosphate homeostasis, but many other so called non-classical actions of 1,25D are known. One of these non-classical actions is, the above mentioned, monocytic differentiation of AML cells [96,97]. In order to separate calcemic properties of 1,25D from other activities of the compound, many low-calcemic vitamin D analogs (VDAs) have been synthesized for various clinical purposes [106-108]. Some VDAs have shown very promising anti-leukemic activities *in vitro* and *in vivo* [109-113]. Encouraged by early observations of 1,25D-induced differentiation of AML cells, few clinical trials were conducted to test the ability of 1,25D to treat myelodysplastic syndrome (MDS) and AML [114,115]. Since hypercalcemia is a limiting factor in clinical use of 1,25D in cancer patients, a low-calcemic analog, paricalcitol was also used to treat MDS in a small clinical trial [116]. Results of these trials were disappointing, therefore at present the use of VDAs in combination therapy is postulated. Several different combinations of drugs for use together with VDAs have been proposed and in most of them the goal is to potentiate differentiating effects of 1,25D or VDAs. This way calcemic effects could be avoided by lowering doses of 1,25D or VDAs necessary to obtain differentiation of leukemic cells. A series of papers has shown that antioxidants, such as carnosic acid, silibinin and curcumin effectively potentiate 1,25D-induced cell differentiation *in vitro* [117-119] and extend the life span of mice inoculated with murine leukemia [110,120]. Similar effect of enhanced differentiation could be obtained using everolismus, immunosuppressant used in transplantation medicine and in oncology, which inhibits mammalian target of rapamycin (mTOR) [121]. Interestingly inhibitors of p38 kinases α and β [122], as well as inhibitors of phospholipase A_2_ [123] and non-specific inhibitors of cyclooxygenase (COX) [124] do the same. All compounds mentioned above share the ability to interfere with 1,25D-induced cell signaling in leukemic cells and, what is important, most of them are accepted drugs. The second strategy is to combine 1,25D or VDAs with agents that trigger cell death, for example with nutlin-3, which enhanced pro-apoptotic and downregulated anti-apoptotic proteins [125]. Many studies have shown that 1,25D potentiates the anti-tumor activities of chemotherapeutics agents [126], predominantly in solid cancers. However, there are also examples of clinical usefulness of 1,25D and cytarabine combination, which prolonged remission in elderly patients with acute AML and MDS [127,128].

Another approach is to select from a great variety of AMLs, only these which are susceptible to 1,25D. A good analogy exists in ATRA treatment, which is very effective in APL patients, but not in other subtypes of AML. It has been shown that individual responses of *ex vivo* cultured blast cells from patients with AML to differentiation-inducing effect of 1,25D are variable [118]. The analysis performed in order to look for correlations between mutations that are often diagnosed in blast cells of AML patients, with the susceptibility of the blasts towards VDA-induced differentiation, has shown that the most susceptible cells are these that carry monosomy 7 or partial loss of 7q [129]. Monosomy 7 or losses in the long arm of this chromosome occur in about 18% of the AML cases [130] and are often connected with prior MDS or with earlier therapy with alkylating agents [131]. Adult patients with this mutation have a very aggressive disease, great susceptibility to infections and poor prognosis [131]. If the correlation found in *ex vivo* cultured blasts could be translated into therapeutic use of VDAs in patients with monosomy 7 or partial loss of 7q, the differentiation-inducing activity of analogs together with their immunostimulating potential [132] might have beneficial effects in this group of patients.

### 5.1. 1,25D Signaling Pathways of Cell Differentiation in Brief

**Figure 2.**
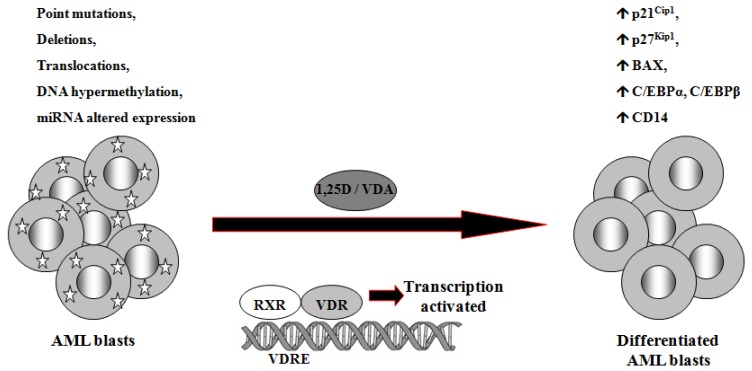
1,25D-induced differentiation of AML cells. AML cells are characterized by a block at various stages of hematopoietic differentiation, that leads to uncontrolled proliferation and accumulation of immature myeloid blast cells in bone marrow and peripheral blood. Differentiation blockage is often caused by various mutations, DNA hypermethylation, as well as miRNA altered expression. AML blasts, when exposed to 1,25D or VDA, acquire features of normal monocytes and become arrested in the G1 phase of the cell cycle. (1,25D: 1,25-dihydroxyvitamin D_3_; VDA: 1,25D analog; RXR: retinoid X receptor; VDR: Vitamin D Receptor; VDRE: vitamin D response elements).

1,25D is one of the steroid hormones that exert biological activity through interaction with their specific nuclear receptors. Vitamin D receptor (VDR) belongs to the same superfamily of nuclear receptors as RAR and RXR. VDR is a ligand-induced transcription factor and a major regulator of 1,25D effects [133]. After ligation with 1,25D or VDAs, VDR becomes protected from degradation [134] and translocates from cell cytosol to the nucleus [135,136]. VDAs with high pro-differentiating potential efficiently stabilize VDR protein and cause its accumulation in the nuclei of the target cells [111]. VDR target genes are connected with the calcium/phosphate homeostasis, but also with anti-proliferative, pro-apoptotic and pro-differentiating actions of 1,25D in non-calcemic tissues. Among such genes are inhibitors of cell cycle, such as p21^Cip1^ and p27^Kip1^ [137], pro-apoptotic Bax [138] and transcription factors of monocytic lineage differentiation, such as C/EBPα and β [139,140]. C/EBPα and β regulate transcription of many downstream genes that encode proteins important for proper macrophage function, such as CD14 cell surface molecule, a component of the innate immune system, acting as a co-receptor of bacterial lipopolysaccharide [139,141]. Regulation of target genes by 1,25D may be obtained not only through transcription. It has been documented that 1,25D-induced increase in p27^Kip1^ at both mRNA and protein levels results from decreased expression of in p27^Kip1^ inhibitors miR181a/b. Forced expression of pre-miR181a in leukemic cells not only abrogated 1,25D-induced increase in p27^Kip1^, but also blunted differentiation effect and reduced cell cycle arrest in 1,25D-treated cells [142]. However, VDR seems not to be crucial in normal hematopoiesis, since VDR null mice do not show major defects in blood cells development [143]. Therefore it is likely that 1,25D and VDAs through upregulation of C/EBPα and β transcription factors, their downstream target genes, and by other 1,25D-dependent mechanisms might bypass normal pathways of myeloid differentiation [144], which are blocked in some leukemic blasts (Figure 2).

## 6. Conclusions

Significant clinical improvement of patients with APL treated with ATRA raised reasonable hope for other agents, such as 1,25D and VDAs, to be effective differentiation-inducing drugs. Further improvements of APL therapy with ASO and detailed understanding of molecular mechanisms of both drugs have shown that therapies may be tailored for specific abnormalities present in neoplastic cells. Novel insights into the etiology of leukemia are of major importance for clinical utility in future drugs [144-146] and may help to select susceptible targets for differentiation therapy using 1,25D and VDAs. It is obvious that the new therapeutic approach should be directed towards leukemic cells, without general cytotoxicity to the organism.
